# Complete Genome Sequence of a Halophilic Bacterium, *Halomonas* sp. Strain NyZ770, from Mariana Trench Sediment

**DOI:** 10.1128/MRA.01037-21

**Published:** 2021-12-09

**Authors:** Xinting Lai, Ying Xu

**Affiliations:** a State Key Laboratory of Microbial Metabolism, Joint International Research Laboratory of Metabolic & Developmental Sciences and School of Life Sciences & Biotechnology, Shanghai Jiao Tong University, Shanghai, China; Montana State University

## Abstract

*Halomonas* sp. strain NyZ770 is a bacterium that was isolated from Mariana Trench sediment. Here, the complete genome sequence of this strain is reported. The genome was sequenced with the Illumina NovaSeq and Pacific Biosciences Sequel sequencing platforms and consists of a single chromosome of 4,024,853 bp, with a G+C content of 60.21%.

## ANNOUNCEMENT

*Halomonas* species are moderately halophilic bacteria that have been reported to be distributed in a variety of saline environments, even deep-sea environments (1, 2). Here, we report the complete genome sequence of *Halomonas* sp. strain NyZ770, a bacterium from the hadal trench, to better understand deep-sea microbiology. *Halomonas* sp. strain NyZ770 was isolated from hadal trench sediment that had been collected at 6,300 m, at a depth of 16 to 20 cm below the seafloor, from the Challenger Deep in the Mariana Trench (10.8895°N, 142.2260°E). The sediment sample was enriched in artificial seawater (ASW) basal medium, a modified ASW medium (3) omitting carbon sources, with 30 mM glucose and 10 mM *N*-acetylglucosamine under *in situ* pressure of 60 MPa for 6 months and then was selectively enriched in ASW basal medium with 1 mM 4-hydroxybenzoate (4HBA) for an additional 4 months. Finally, serial dilution on ASW agar plates with 4HBA under ambient pressure (0.1 MPa) yielded an isolate designated strain NyZ770. The 16S rRNA gene from strain NyZ770 exhibited the greatest identity (>99%) to those from three strains of the genus *Halomonas* (GenBank accession numbers AB362301.1, MN258543.1, and CP023656.1), indicating that it should be classified as a *Halomonas* strain.

For genomic sequencing, a single colony of strain NyZ770 was cultured overnight in liquid lysogeny broth (LB) (4, 5) before the cells were washed twice and harvested. The genomic DNA was extracted by using the cetyltrimethyl ammonium bromide (CTAB) (6) method with minor modifications. The NovaSeq library was prepared using the TruSeq DNA sample preparation kit (Illumina, USA) with an insert size of 400 bp, in paired-end (2 × 150-bp) sequencing mode. The Pacific Biosciences (PacBio) sequencing library was prepared using the template preparation kit v1.0 (PacBio, USA) with an insert size of 20 kb, in standard sequencing mode. Genome sequencing was performed by Personal Biotechnology Co. (Shanghai, China) using the Illumina NovaSeq and PacBio Sequel platforms.

For the Illumina data, the number of reads is 8,702,654 reads in total. For the PacBio data, the read *N*_50_ is 11,406 bp, with a total sequence number of 245,204 sequences. AdapterRemoval (v2.2.2) (7) and SOAPec (v2.03) (8) were used to remove the joint contamination and to filter the error reads, respectively. The resulting data from the PacBio sequencing platform were assembled with HGAP (v4) (9) and Canu (v1.7.1) (10) software to obtain the contig sequence. The Illumina data were used by Pilon (v1.18) (11) to correct the resulting PacBio assembly. For the circularization step, the overlap was identified and trimmed with Circlator (v1.5.5) (12). GeneMarkS (v4.32) (13) was used for gene prediction for the complete genome. tRNAscan-SE (v1.3.1) (14) was used to predict tRNAs, and Barrnap software (v0.9) (15) was used to predict rRNAs (5S rRNAs, 16S rRNAs, and 23S rRNAs). Default parameters were used for all software unless otherwise noted. The protein-coding genes were annotated with the NCBI Non-Redundant Protein Sequence (NR), Kyoto Encyclopedia of Genes and Genomes (KEGG), eggNOG, and Swiss-Prot databases.

The complete genome of this strain consisted of a single chromosome of 4,024,853 bp, with a G+C content of 60.21%. The number of open reading frames (ORFs) was 3,690, the total ORF length was 3,515,844 bp, the average ORF length was 952.80 bp, the ORF/genome value (coding percentage) was 87.35%, and the G+C content in the ORF region was 61.12%. Genome annotation suggested that strain NyZ770 has 3,542, 2,174, and 2,790 protein-coding genes according to the NR, KEGG, and Swiss-Prot databases, respectively, which accounted for 95.99%, 58.92%, and 75.61% of the total number of predicted ORFs, respectively. Regarding noncoding RNA prediction, the tRNA, 5S rRNA, 16S rRNA, and 23S rRNA copy numbers were 60, 6, 6, and 6, respectively.

This assembly will facilitate genome-wide comparison studies with a focus on the ecology of bacteria living in hadal trench environments under high hydrostatic pressures.

### Data availability.

The genome sequence and annotation data for *Halomonas* sp. strain NyZ770 were deposited in GenBank under BioProject number PRJNA769991, BioSample number SAMN22187422, and GenBank accession number CP085143. The raw Illumina and PacBio data for strain NyZ770 were deposited in the Sequence Read Archive (SRA) under BioProject number PRJNA769991 and SRA accession number SRS11013316.
